# Survival benefit of radical prostatectomy in patients with advanced TURP-diagnosed prostate cancer: a population-based real-world study

**DOI:** 10.1186/s12893-024-02430-2

**Published:** 2024-05-03

**Authors:** Deng Lin, Le Lin, Liefu Ye, Tao Li, Yongbao Wei, Lizhi Li

**Affiliations:** 1 Shengli Clinical Medical College of Fujian Medical University, Fuzhou, 350001 China; 2 Department of Urology, Fujian Provincial Hospital South Branch, Fuzhou, China; 3 Department of Urology, Fujian Provincial Hospital, Fuzhou, Fujian 350001 China; 4 Department of Pediatric Surgery, Fujian Provincial Hospital, Fuzhou, 350001 China

**Keywords:** Prostate cancer, TURP, Radical prostatectomy, Survival benefit, Elderly, Marching study

## Abstract

**Objectives:**

A considerable number of patients are diagnosed with prostate cancer (PCa) by transurethral resection of the prostate (TURP). We aimed to evaluate whether radical prostatectomy (RP) brings survival benefits for these patients, especially in the elderly with advanced PCa.

**Patients and methods:**

We used the Surveillance, Epidemiology, and End Results (SEER) database to obtain PCa cases diagnosed with TURP. After the propensity matching score (PSM) for case matching, univariate, multivariate, and subgroup analyses were performed to investigate whether RP impacts the survival benefit.

**Results:**

4,677 cases diagnosed with PCa by TURP from 2010 to 2019 were obtained, including 1,313 RP patients and 3,364 patients with no RP (nRP). 9.6% of RP patients had advanced PCa. With or without PSM, cancer-specific mortality (CSM) and overall mortality (OM) were significantly reduced in the RP patients compared to the nRP patients, even for older (> 75 ys.) patients with advanced stages (all *p* < 0.05). Except for RP, younger age (≤ 75 ys.), being married, and earlier stage (localized) contributed to a significant reduction of CSM risk (all *p* < 0.05). These survival benefits had no significant differences among patients of different ages, married or single, and at different stages (all p for interaction > 0.05).

**Conclusions:**

Based on this retrospective population-matched study, we first found that in patients diagnosed with PCa by TURP, RP treatment may lead to a survival benefit, especially a reduction in CSM, even in old aged patients (> 75 ys.) with advanced PCa.

**Supplementary Information:**

The online version contains supplementary material available at 10.1186/s12893-024-02430-2.

## Introduction

Prostate biopsy is the gold standard for the diagnosis of prostate cancer (PCa) [1, 2], even under the premise of the current advances in imaging technology [3]. Transurethral resection of the prostate (TURP) is not recommended for its diagnostic purposes only; however, for patients with suspected PCa with obstructive symptoms, TURP may be considered as a treatment option [1, 4]. It is related to the efficacy of TURP in relieving bladder outlet obstruction [5, 6]. In clinical practice, many patients diagnosed with PCa are diagnosed by TURP [7]. The incidental PCa (iPCa) rate was low in patients receiving TURP without biopsy diagnosis, about 8%, and the vast majority were localized PCas with early stage [8]. In the United States, iPCa is mainly diagnosed in men younger than 80 years of age [9], as many of them were not recommended for PCa screening [10], but these patients may have a longer life expectancy and may require follow-up treatment for PCa. The optimal treatment after TURP diagnosis, such as whether radical prostatectomy (RP) once remained controversial, and the survival prognosis was not well reported [7, 11]. A recent retrospective study in Denmark reported survival in patients with iPCa; they included 64,059 patients with TURP, 63,781 with a final diagnosis of PCa, 42,558 of whom were not screened for biopsy, and they found that these patients had a shallow risk of PCa-specific death, the 15-year cumulative incidence of all patients was 1.4% [7]. However, this study only analyzed the survival of PCa patients diagnosed with TURP and did not study the effect of RP after TURP on survival; their patients had generally localized stages of PCa, and the prognosis of advanced PCa remained unknown. A systematic review and meta-analysis published in 2019 analyzed 15 studies of patients with RP after TURP (*n* = 6,840) and found that RP after prior TURP resulted in worse perioperative, oncological, and functional outcomes [12]. Unfortunately, none of these studies included an analysis of the survival benefit of RP in these patients. Similarly, studies in the past three years focused on the adverse effects of TURP on RP, including perioperative, functional, and oncological outcomes, and had not paid attention to the effect of RP after TURP on survival benefit [13–17].

Indeed, the survival benefit of radical local therapy, including RP or radical radiotherapy (RT) for localized prostate cancer, is well known [1, 18]; its survival benefit even exists in selective patients with lymph node metastasis or distant metastasis [19, 20]. However, these conclusions were generally based on biopsy-confirmed PCa. For PCa patients diagnosed with TURP, whether RP or RT brought survival benefits seemed to be beneficial in theoretical reasoning. There is a lack of robust data to support this reasoning conclusion. Especially for progressive PCa, it wasn’t easy to speculate whether RP brought survival benefits after TURP. Thus, our study intended to investigate the effect of RP on survival, including PCa-specific mortality (CSM) and overall mortality (OM), in patients with PCa who had not undergone biopsy but had been confirmed PCa by TURP to guide future clinical practice.

## Methods

We obtained data access and downloaded data from the Surveillance, Epidemiology, and End Results (SEER) database. Data on PCa patients recorded in the database from 1975 to 2019 were obtained in October 2022. Only all cases with PCa diagnosed by TURP were included, and patients with PCa who had undergone biopsy were not included. Data were then excluded according to the following exclusion criteria: (a) survival time less than one month, (b) CSM unknown, (c) age greater than 90 years or less than 20 years. The patient’s cancer stage was based on the records of the summary stage according to the SEER database 2004. It was divided into localized, regional, and distant stages. We defined death for CSM as PCa-specific death, while patients with alive or other death were defined as a non-CSM event. OM was defined in two opposite results: the patient dead or alive.

Data was processed using the SPSS package (SPSS 27.0 for Windows; SPSS Inc.). Continuous variables, including age (years old = ys.), month to treatment (month = mo.), and survival time (mo.), were represented by median, quartile, and range. Two-sample nonparametric tests were used to compare the parameters of the RP and no RP (nRP) groups. It should be noted that for patients in the nRP group, this only signified that these individuals did not undergo prostatectomy; however, a minority of patients might have received other cancer-directed surgeries, primarily aimed at metastatic lesions; especially for those whose primary tumors were inoperable or had no tumor activity following radiotherapy or medication therapy, the sole removal of oligometastatic lesions might be also a clinical method to improve patient survival or quality of life. Then, with 0.002 as the matching tolerance, age, race, marital status, annual income, home location, diagnosis year, stage, and month to treatment were matched according to a 1:1 ratio using propensity matching score (PSM) study. We used the Multivariable Cox proportional hazard model to set up two models, namely adjusted model 1 (adjusted for age, race, and stage) and adjusted model 2, which was adjusted for age, race, marital status, annual income, home location, diagnosis year, stage, month to treatment, and cancer-directed surgery (CDS). Then the age after PSM was divided into two groups according to the median 75ys; the race was divided into two groups according to white or not; the stage was divided into two groups: localized, advanced (regional and distant). Together with other parameters (RP and nRP, marital status, annual income, home location, diagnosis year, radiation therapy, and systemic therapy), the Kaplan–Meier method and Cox regression analysis were used to evaluate the impact of each quantity on CSM and OM, respectively. A subgroup analysis of related variables was further performed. The hazard ratio (HR) and 95% confidence interval (CI) and their *p*-values ​​were calculated. A *p*-value less than 0.05 was considered statistically significant.

## Results

### Comparison of two groups of patients with RP and nRP before PSM

**Fig. 1:**
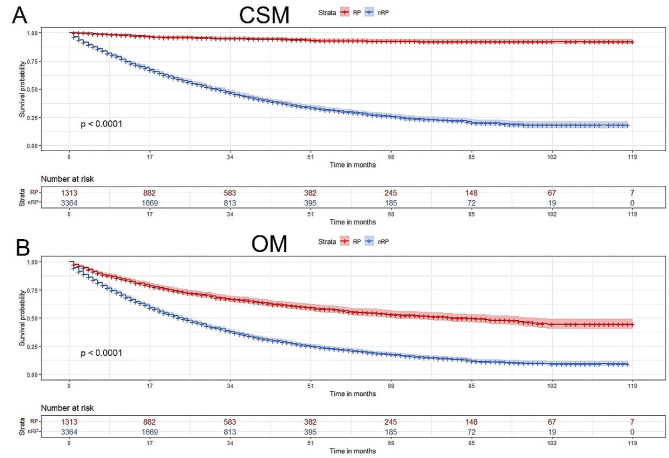
CSM and OM for patients diagnosed PCa by TURP from 2010 to 2019. (**A**) RP patients (*n* = 1,313) have lower rates of CSM compared with nRP patients (*n* = 3,364); (**B**) RP patients have lower rates of OM compared with nRP patients (both *p* < 0.001) Cancer-specific survival; OM = overall survival; RP radical prostatectomy; TURP transurethral resection of the prostate

We finally obtained 4,677 cases for this study. All TURP-diagnosed PCa patients were from 2010 to 2019, including 1,313 cases in the RP group and 3,364 in the nRP group. We found that 65.9% (*n* = 865) of RP patients had postoperative pathological Gleason score (GS) 6, 29.8% (*n* = 391) GS 7, and 4.3% (*n* = 57) GS 8–10. These RP patients had 90.4% (*n* = 1,187) localized stage of disease, and 9.6% had advanced PCa (8.5% regional stage and 0.5% distant stage). Compared with the nRP group, the median patient age in the RP group was younger (70.00ys. vs. 75.00ys.), more percentage of white race (89.3% vs. 72.4%), more got married (68.6% vs. 51.7%), earlier percentage of diagnosis year (2010–2014 45.3% vs. 39.2%), lower prostate-specific antigen (PSA) (98.0 ng/ml or greater: 0.5% vs. 80.4%), more percentage of localized stage (90.4% vs. 4.8%), more percentage of lymph node dissection (4 or more regional lymph nodes: 76.6% vs. 0.1%), less percentage of systemic therapy (2.3% vs. 10.6%), lower rates of CSM (4.6% vs. 49.8%) and OM (37.5% vs. 64.0%), and had longer survival time (30.00 mo. vs. 16.00 mo.) (all *p* < 0.001) (Table 1; Fig. 1).

**Table 1:** Comparisons between TURP-diagnosed PC patients with RP and nRP

Variables	RP (*n* = 1,313)		nRP (*n* = 3,364)		*P* value
	*N*	%	*N*	%	
Age (ys.)					< 0.001
Median	70.00		75.00		
IQR (range)	63.00–77.00 (41–90)		65.00–83.00 (24–90)		
Race					< 0.001
White	1,173	89.3	2,434	72.4	
Black	71	5.4	578	17.2	
Others	67	5.1	341	10.1	
Missing	2	0.2	11	0.3	
Marital status					< 0.001
Married	901	68.6	1,739	51.7	
Single	331	25.2	1,414	42.0	
Missing	81	6.2	211	6.3	
Annual income					0.07
< 7, 000$	659	50.2	1,591	47.3	
≥ 7,000$	654	49.8	1,772	52.7	
Missing			1	0.0	
Home location					< 0.001
Big city	585	44.6	1,829	54.4	
Small city	728	55.4	1,534	45.6	
Missing			1	0.0	
Diagnosis year					< 0.001
2010–2014	595	45.3	1,319	39.2	
2014–2019	718	54.7	2,045	60.8	
PSA					< 0.001
0.1 or less nanograms/milliliter (ng/ml)	11	0.8	6	0.2	
Test ordered, results not in the chart	29	2.2	84	2.5	
98.0 ng/ml or greater	6	0.5	2,704	80.4	
Missing	1,267	96.5	570	16.9	
Pathological Gleason score					/
≤ 6	865	65.9	/	/	
7	391	29.8		/	
8–10	57	4.3	/	/	
Stage					< 0.001
Localized	1,187	90.4	163	4.8	
Reginal	111	8.5	126	3.7	
Distant	7	0.5	2,900	86.2	
Missing	8	0.6	175	5.2	
Month to treatment (mo.)					< 0.001
N	1,311	99.8	2,752	81.8%	
Median	0.00		0.00		
IQR (range)	0.00–0.00 (0.00–12.00)		0.00–1.00 (0.00–23.00)		
Missing	2	0.2	612	18.2%	
CDS					< 0.001
Yes	1,313	100	100	3.0	
No	0	0	3,186	94.7	
Missing			78	2.3	
Lymph node dissection					< 0.001
1 to 3 regional lymph nodes removed	65	5.0	14	0.4	
4 or more regional lymph nodes removed	1,006	76.6	5	0.1	
Biopsy only	0	0	127	3.8	
Missing	242	18.4	3,218	95.7	
Radiation therapy					0.72
Beam	8	0.6	728	21.6	
Others			12	0.4	
No/known	1305	99.4	2,624	78.0	
Chemotherapy					< 0.001
Yes	17	1.3	435	12.9	
No/unknown	1,296	98.7	2,929	87.1	
Systemic therapy					< 0.001
In and before surgery	4	0.3	52	1.5	
After surgery	24	1.8	306	9.1	
No/ unknown	1,285	97.9	3,006	89.4	
CSM					< 0.001
Death	60	4.6	1,674	49.8	
Alive or other death	1,253	95.4	1,690	50.2	
OM					< 0.001
Death	493	37.5	2,154	64.0	
Alive	820	62.5	1,210	36.0	
Survival time (mo.)					< 0.001
Median	30.00		16.00		
IQR (range)	12.00–58.00 (1-119)		7.00–33.00 (1-118)		

TURP = transurethral resection of the prostate; CDS = cancer-directed surgery; CSM = cancer-specific survival; OM = overall survival; RP = radical prostatectomy; nRP = no radical prostatectomy; IQR = interquartile range

### Comparison of two groups of patients with RP and nRP after PSM

**Fig. 2:**
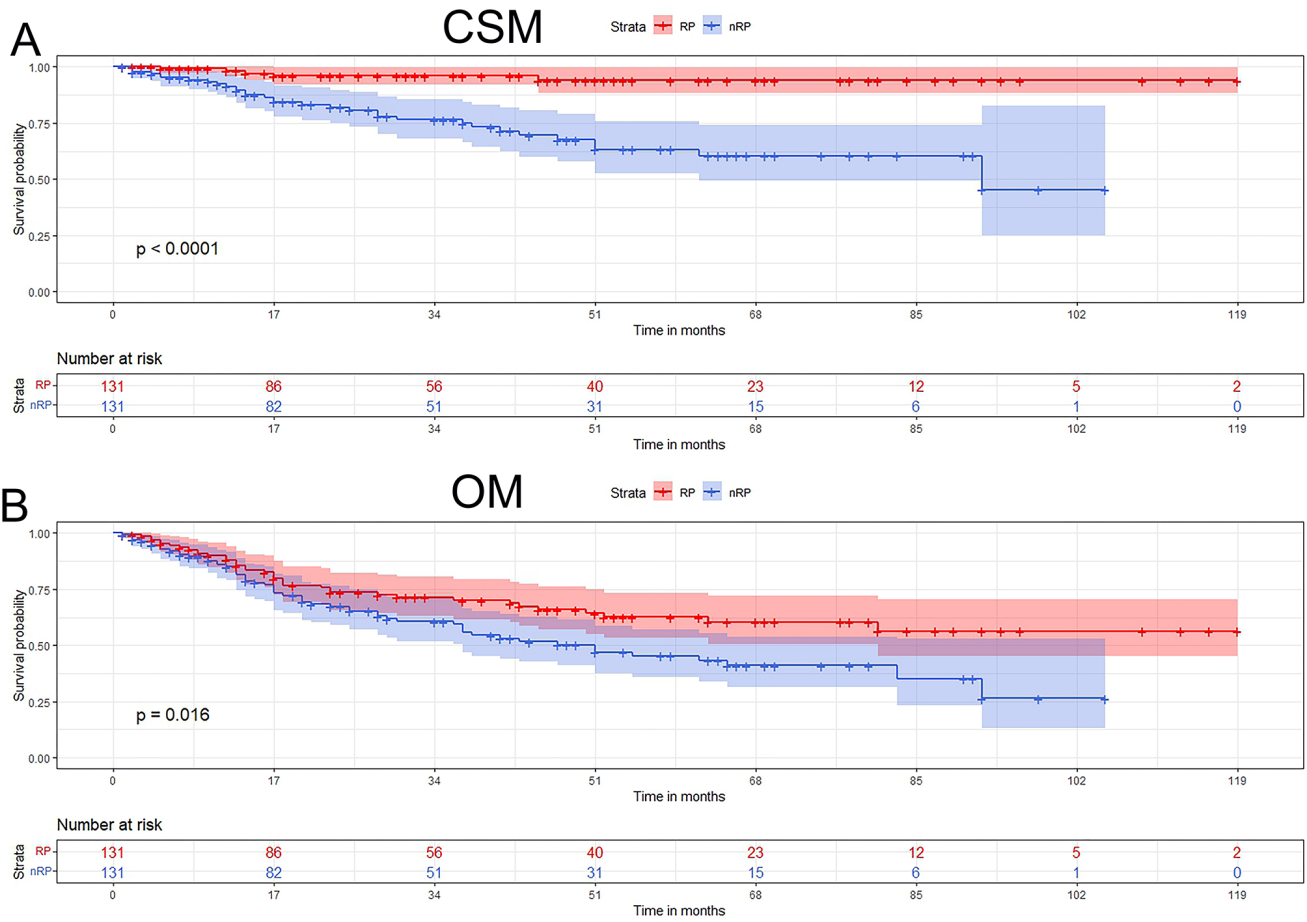
CSM and OM for patients diagnosed PCa by TURP from 2010 to 2019 after PSM. (**A**) RP patients (*n* = 131) have lower rates of CSM compared with nRP patients (*n* = 131) (*p* < 0.001); (**B**) RP patients have lower rates of OM compared with nRP patients (*p* < 0.05) PSM = propensity score matching (by1:1 matching); CSM = cancer-specific survival; OM = overall survival; RP = radical prostatectomy; nRP = no radical prostatectomy; TURP = transurethral resection of the prostate

After PSM, a total of 232 cases were obtained. There were 131 patients with RP and nRP each. RP patients had 46.6% (*n* = 61) postoperative pathological GS, 42.0% (*n* = 55) GS 7, 11.5% (*n* = 15) GS 8–10. Among these RP patients, only 64.1% (*n* = 84) were localized PCas, and 35.9% were advanced PCas (30.5% regional stage and 5.3% distant stage). Except for PSA, which was not included in PSM due to too many missing values, we found that the pre-treatment data of the two groups were well-matched. There was no statistical difference in the following items, including median age (RP vs. nRP = 74.00 ys. vs. 76.00 ys.), race, marital status, annual income, home location, diagnosis year, stage, and month to treatment (all *p* > 0.05). While RP patients still had a higher proportion of lymph node dissections (4 or more regional lymph nodes removed: 77.1% vs. 0.8%), lower rates of systemic therapy (9.9% vs. 19.1%), lower rates of CSM (3.8% vs. 24.4%) and OM (29.8% vs. 44.3%) (all *p* < 0.05) (Table 2) (Fig. 2). The two groups had no significant difference in radiotherapy, chemotherapy, and median survival time (all *p* > 0.05).

**Table 2:** Comparisons between TURP-diagnosed PC patients with RP and nRP after PSM

Variables	RP (*n* = 131)		nRP (*n* = 131)		*P* value
	*N*	%	*N*	%	
Age (ys.)					0.08
Median	74.00		76.00		
IQR (range)	67.00–79.00 (53–90)		66.00–82.00 (47–90)		
Race					0.32
White	104	79.4	111	84.7	
Black	14	10.7	7	5.3	
Others	13	9.9	12	9.2	
Missing	0	0	1	0.8	
Marital status					0.83
Married	87	66.4	87	66.4	
Single	33	25.2	38	29.0	
Missing	11	8.4	6	4.6	
Annual income					0.71
< 7, 000$	65	49.6	62	47.3	
≥ 7,000$	66	50.4	69	52.7	
Home location					0.22
Big city	71	54.2	61	46.6	
Small city	60	45.8	70	53.4	
Diagnosis year					1
2010–2014	49	37.4	49	37.4	
2014–2019	82	62.6	82	62.6	
PSA					< 0.001
0.1 or less nanograms/milliliter (ng/ml)	3	2.3	0	0	
Test ordered, results not in the chart	5	3.8	6	4.6	
98.0 ng/ml or greater	4	3.1	49	37.4	
Missing	119	90.8	76	58.0	
Pathological Gleason score					/
≤ 6	61	46.6	/	/	
7	55	42.0		/	
8–10	15	11.5	/	/	
Stage					0.71
Localized	84	64.1	82	62.6	
Reginal	40	30.5	39	29.8	
Distant	7	5.3	10	7.6	
Month to treatment (mo.)					0.20
Median	0.00		0.00		
IQR (range)	0.00–0.00 (0.00–12.00)		0.00–1.00 (0.00–2.00)		
CDS					< 0.001
Yes	1313	100	59	45.0	
No	0	0	71	54.2	
Missing			1	0.8	
Lymph node dissection					< 0.001
1 to 3 regional lymph nodes removed	5	3.8	3	2.3	
4 or more regional lymph nodes removed	101	77.1	1	0.8	
Biopsy only	0	0	10	7.6	
Missing	25	19.1	117	89.3	
Radiation therapy					0.64
Beam	1	0.8	13	9.9	
Others	0	0	3	2.3	
No/known	130	99.2	115	87.8	
Chemotherapy					0.58
Yes	6	4.6	8	6.1	
No/known	125	95.4	123	93.9	
Systemic therapy					< 0.05
In and before surgery	3	2.3	2	1.5	
After surgery	10	7.6	23	17.6	
No/known	118	90.1	106	80.9	
CSM					< 0.001
Death	5	3.8	32	24.4	
Alive or other death	126	96.2	99	75.6	
OM					< 0.05
Death	39	29.8	58	44.3	
Alive	92	70.2	73	55.7	
Survival time (mo.)					0.32
Median	28.00		24.00		
IQR (range)	12.00–54.00 (1-119)		10.00–49.00 (1-105)		

TURP = transurethral resection of the prostate; CDS = cancer-directed surgery; CSM = cancer-specific survival; OM = overall survival; RP = radical prostatectomy; nRP = no radical prostatectomy; IQR = interquartile range

### RP brought survival benefits in PCa patients compared with nRP

Comparing nRP patients, regardless of whether the data were treated before or after PSM, RP significantly reduced risks of CSM and OM (all *p* < 0.05) in both the non-adjusted model and adjusted model 1. Among them, RP reduced risks of CSM with HRs of 0.21 (95%CI 0.15–0.30) (before PSM) and 0.13 (95%CI 0.43 − 0.37) (after PSM) (both *p* < 0.001), indicating RP declined CSM risk by 79% and 87, respectively in adjusted model 1. RP also brought about a reduction in OM, with HRs 0.77 (95%CI 0.63–0.93) (before PSM) and 0.62 (95%CI 0.38–0.99) (after PSM) (both *p* < 0.05), in which OM risk was reduced by 23% and 38% respectively in adjusted model 1. In adjusted model 2, only a significant reduction in CSM was observed in RP patients, with HRs of 0.34 (95%CI 0.19–0.60) (before PSM) and 0.12 (95%CI 0.03–0.57) (after PSM) (both *p* < 0.01), and the risk of CSM was reduced by 66% and 88%, respectively. However, no significant change in the risk of OM was observed (both *p* > 0.05) In adjusted model 2 (Table 3). Our results suggested that RP may have an advantage in reducing the risk of CSM but had no significant effect on the risk of OM in adjusted model 2.

**Table 3:** Multivariable Cox proportional hazard model for CSM and OM for RP patients diagnosed PCa by TURP

Outcomes	RP HR (95% CI)	*P*-value
CSM		
Non-adjusted	0.06 (0.05–0.08)	< 0.001
Adjusted model 1	0.21 (0.15–0.30)	< 0.001
Adjusted model 2	0.34 (0.19–0.60)	< 0.001
PSM non-adjusted	0.14 (0.06–0.36)	< 0.001
PSM Adjusted model 1	0.13 (0.04–0.37)	< 0.001
PSM Adjusted model 2	0.12 (0.03–0.57)	< 0.01
OM		
Non-adjusted	0.39 (0.36–0.43)	< 0.001
Adjusted model 1	0.77 (0.63–0.93)	< 0.01
Adjusted model 2	1.19 (0.83–1.71)	0.35
PSM non-adjusted	0.61 (0.41–0.92)	< 0.05
PSM Adjusted model 1	0.62 (0.38–0.99)	< 0.05
PSM Adjusted model 2	0.69 (0.34–1.41)	0.31

Adjusted model 1 adjusts for age, race, and stage

Adjusted model 2 adjusts for age, race, marital status, annual income, home location, diagnosis year, stage, month to treatment, and CDS.

The PSM-non-adjusted model adjusts for none

PSM-adjusted model 1 adjusts for age, race, and stage

PSM-adjusted model 2 adjusts for age, race, marital status, annual income, home location, diagnosis year, stage month to treatment, and CDS.

HR = hazard ratio; PSM = propensity score matching (by1:1 matching); CDS = cancer-directed surgery performed; CI = confidence interval; CSM = cancer-specific survival; OM = overall survival; RP = radical prostatectomy; nRP = no radical prostatectomy; TURP = transurethral resection of the prostate

### Univariate, multivariate, and subgroup analysis of other influencing factors on CSM and OM

We further analyzed the effect of other factors on CSM and OM after PSM. For CSM, we found that except RP had a benefit in CSM risk reduction, younger age (≤ 75 ys.) (HR = 0.30, 95%CI 0.15–0.63), being married (HR = 0.36, 95%CI 0.19–0.71), and earlier stage (localized) (HR = 0.42, 95%CI 0.19–0.94) were all helpful For CSM risk reduction. In addition to RP bringing about risk reduction of OM, only younger age (≤ 75 ys.) (HR = 0.51, 95%CI 0.34–0.78) and earlier stage (localized) (HR = 0.65, 95%CI 0.43–0.98), contributed to OM risk reduction (both *p* < 0.05). While race, annual income, home location, radiation therapy, and systemic therapy had no significant effect on CSM and OM risk reduction (all *p* > 0.05).

Subgroup analysis found that patients of different ages (≤ 75 ys. or > 75 ys.) and different marital statuses and different stages could have a reduced risk of CSM from RP (all *p* < 0.05), which suggested that even for older patients (age > 75 ys.) with advanced PCa (regional and distant stages), a reduction in the risk of CSM may be obtained when RP offered (Table 4). Interestingly, only patients aged > 75 ys. or with advanced PCa had a reduced risk of OM from RP (all *p* < 0.05) (Table 5). It was further found that patients with RP had no significant difference in the risk of CSM between different ages, marital statuses, and stages (all p for interaction > 0.05). Similarly, there was no significant difference in the risk of OM among RP patients between different age groups and different stages (both p for interaction > 0.05). It should be noted that in our analysis mentioned above, aside from RP, factors such as age, marital status, and disease stage may also be key determinants influencing CSM) and/or OM. However, through subgroup analysis, no intergroup differences in CSM and OM were observed among different age, marital status, and disease stage subgroups. The inconsistency between these two conclusions was, in fact, not contradictory. This could be related to the sample size of each subgroup, the method of subgroup classification, etc.

**Table 4:** Results of univariate, multivariate analyses and subgroup analysis for CSM after PSM

Variables		Univariate *p*-value	Multivariate						Subgroup analysis
			HR	95% CI	*p*-value	HR	95% CI	*p*-value	p for interaction between values and RP
Age		< 0.05	0.30	0.15–0.63	< 0.01				0.86
	≤ 75 ys.					0.17	0.04–0.77	< 0.05	
	> 75 ys.					0.14	0.04–0.46	< 0.01	
RP		< 0.001	0.14	0.05–0.36	< 0.001				
	RP								
	nRP								
Race		0.71							
	White								
	Others								
Marital status		< 0.05	0.36	0.19–0.71	< 0.01				0.44
	Married					0.10	0.02–0.43	< 0.05	
	Single					0.24	0.07–0.82	< 0.05	
Annual income		0.16							
	< 7000$								
	≥ 7000$								
Home location		0.09							
	Big city								
	Small city								
Diagnosis year		0.23							
	2010–2014								
	2015–2019								
Stage		< 0.01	0.42	0.19–0.94	< 0.05				0.41
	Localized					0.07	0.01–0.50	< 0.01	
	Regional and distant					0.12	0.06–0.54	< 0.01	
Radiation therapy		0.68							
	Yes								
	No/unknown								
Systemic therapy		< 0.01	2.13	0.89–5.02	0.09				
	Yes								
	No/unknown								

HR = hazard ratio; PSM = propensity score matching (by1:1 matching); CI = confidence interval; CSM = cancer-specific survival; RP = radical prostatectomy; nRP = no radical prostatectomy

**Table 5:** Results of univariate, multivariate analyses and subgroup analysis for OM after PSM

Variables		Univariate *p*-value	Multivariate						Subgroup analysis	
			HR	95% CI	*p*-value	HR	95% CI	*p*-value		p for interaction between values and RP
Age		< 0.001	0.51	0.34–0.78	< 0.01					0.52
	≤ 75 ys.					0.76	0.40–1.43	0.39		
	> 75 ys.					0.56	0.33–0.95	< 0.05		
RP		< 0.05	0.59	0.39–0.91	< 0.05					
	RP									
	nRP									
Race		0.11								
	White									
	Others									
Marital status		< 0.05	0.68	0.44–1.04	0.08					
	Married									
	Single									
Annual income		0.91								
	< 7000$									
	≥ 7000$									
Home location		0.66								
	Big city									
	Small city									
Diagnosis year		0.67								
	2010–2014									
	2015–2019									
Stage		< 0.05	0.65	0.43–0.98	< 0.05					0.22
	Localized					0.76	0.44–1.31	0.32		
	Regional and distant					0.43	0.23–0.81	< 0.01		
Radiation therapy		0.56								
	Yes									
	No/unknown									
Systemic therapy		0.49								
	Yes									
	No/unknown									

HR = hazard ratio; PSM = propensity score matching (by1:1 matching); CI = confidence interval; OM = overall survival; RP = radical prostatectomy

## Discussion

We used a retrospective study to find that PCa patients diagnosed with TURP, regardless of their age or marital status, could benefit from subsequent treatment of RP, especially with a significant reduction in the risk of CSM, even in old (> 75ys.) patients with advanced PCa. The conclusions of this study may provide a reference for subsequent treatment options for PCa patients diagnosed with TURP. Our findings differed from those of iPCas in a previous study [7]; we included many patients with later stages. Thus, our findings may be more helpful for advanced PCa patients diagnosed with TURP to choose subsequent treatment options, such as RP.

TURP could supplement the diagnosis of PCa to a certain extent and could relieve the symptoms of obstruction at the same time [21]. For benign patients diagnosed by TURP with PSA < 10 ng/ml, the cumulative incidence of future PCa was very low, only about 3% [7]. However, from the perspective of diagnosing PCa, it appeared that TURP had a limited role. Growing evidence indicated that TURP might be inferior to Holmium laser enucleation of the prostate (HoLEP) for PCa diagnosis [22], especially in patients with negative preoperative biopsy, it was not uncommon for HoLEP to confirm PCa (5.64%, 70/1240) [23]. But even so, neither TURP nor HoLEP could replace the golden status of biopsy in diagnosing PCa [1, 2]. Although our study included many TURP-diagnosed PCa patients, we believed that TURP was primarily based on improvement in patients’ obstructive symptoms, with the diagnosis of PCa as a secondary objective. We did not advocate TURP based on the purpose of diagnosis. For PCa patients who chose TURP first, RP was a promising choice of subsequent treatment and may bring survival benefits based on our study.

TURP is not a curative procedure for PCa; residual cancer was found in RP specimens from 67% (77/153) of early-stage patients who had undergone prostate endo-surgery such as TURP [24]. Inconsistency of the GS before and after RP was typical due to RP after TURP, with 30% of patients (*n* = 104) having an upgraded GS and 42% showing degraded or no residual tumor [25]. Therefore, it brought specific difficulties to the treatment of PCa after diagnosis. We suggested that PCa patients diagnosed with TURP may refer to the risk stratification of current guidelines or consensus recommendations for PCa patients confirmed by biopsy [1, 2, 19]. Like patients undergoing HoLEP, active surveillance was generally considered for low- and intermediate-risk PCa patients after HoLEP. At the same time, local and systemic therapy should be recommended for high-risk PCa patients [26]. The subsequent treatments after TURP may differ from HoLEP, which may remove more prostate tissue [22]; therefore, it may be helpful for the excellent control of localized PCa, but positive management, such as radical local therapy, should be considered for advanced high-risk PCa. Our study supported RP as a radical local therapy that could bring survival benefits to PCa patients diagnosed with TURP. The survival benefit of HoLEP and the effect of subsequent RP on these PCa patients diagnosed with HoLEP remain to be further studied.

TURP may have little effect on the difficulty of subsequent RP, but it may affect the recovery of sexual function and urinary continence [13, 14]. A previous study suggested that laparoscopic RP was more difficult after TURP than those without TURP [27]. In a recent comparative study, if robot-assisted RP was performed by experienced surgeons, prior transurethral resection or laser enucleation of the prostate did not negatively affect operative, complication-related, and oncological outcomes, including biochemical recurrence or progression of metastases; however, patients had urethrectomy or laser enucleation of the prostate negatively affects erectile function and incontinence recovery [13]. Similar to the previous study, the rate of positive margins and biochemical recurrence in laparoscopic RP surgery after TURP (*n* = 55) was comparable when compared with patients without TURP (*n* = 55) after two years of follow-up [27]. While another study found negative results, robot-assisted prostatectomy after endoscopic surgery for BPH (30/310) was safe and effective, with similar success rates and complication rates as patients who had not undergone endoscopic surgery (280/310), including functional recovery, quality of life, perioperative and post [17]. There was also a study that suggested that the general quality of life score was significantly lower after RP after previous benign prostate surgery [14].

The survival benefit of radical local therapy (RT or RP) for localized prostate cancer is well known [1, 18], even for selective patients with metastasis [19, 20]. Patients with confirmed PCa may also undergo focal therapy, which was a treatment modality for eliminating local cancer tissue, including high-intensity focused ultrasound, cryotherapy, focal laser ablation, and vascular-targeted photodynamic therapy, etc.; previous studies found focal therapy had specific effects on PSA reduction, failure-free survival, recurrence-free survival, and progression-free survival, but whether it could improve CSM and OM remained inconclusive [18, 28]. A recent systematic review found that brachytherapy boosts combined with external beam radiotherapy may be considered for unfavorable nonmetastatic PCa in patients with good urinary function. Still, this recommendation is weak based on the European Urological Association guideline approach [29]. For advanced PCa, although RP also provides survival benefits, it seemed that treatment providers preferred to choose RT [19, 28, 30]. Of the panelists who voted for radical local therapy for advanced PCa, 87% preferred RT to the primary tumor, and only 13% preferred RP [19]. This significant difference in voting ratios may be primarily based on the similar benefit of RT compared to RP, while RT led to lower functional impairment complications [1, 28]; however, it could not be ruled out that there was a particular relationship with too few urologists participating in the voting [19]. Our study found that RP after TURP resulted in an all-patient survival benefit, especially a significant reduction in the risk of CSM, including advanced PCa patients with older age (> 75ys.). However, we emphasized that RP was only a part of multimodal treatment for advanced PCa patients, and multidisciplinary team and systemic therapy were necessary [19].

A study found that benign prostatic hyperplasia (BPH) may increase the risk of death in PCa patients, and even the risk of death increased by 2–8 times [31]. 10-year PCa mortality after TURP in BPH patients was 1.37 (0.81–2.29) [32]. At the same time, it was unclear whether the increased mortality was due to TURP or BPH. However, it did not seem to affect the conclusion of our study. The patients we included were all TURP-treated patients, so the contribution of BPH or TURP to mortality should be the same between the two groups. Furthermore, we focused on the necessity of subsequent treatment after TURP. Our results suggested that RP could significantly reduce the risk of CSM by 66%, and this kind of benefit was beneficial for patients with different ages, marital statuses, and tumor stages; even survival benefit was found in older (> 75ys.) patients with advanced disease (regional and distant stages).

This study had some limitations. The main limitation was that this was a retrospective study. Further prospective trials are needed to validate these findings. Secondly, the data only covered part of North America. Due to the excessive number of missing values, this study did not match PSA, GS, and the number of lymph node dissections, which may also affect the survival outcomes. For example, cases lacked detailed records of pre-surgery PSA levels for the majority of patients undergoing RP, while indeed, this limitation may affect our results regarding survival outcomes. In addressing the missing GS data for patients diagnosed with PCa via TURP in nonRP cohort, it is important to clarify that this limitation arises from the recording practices of the database used. Specifically, GS is only recorded for patients who have undergone needle biopsy. Herein, we highlighted this point to ensure that readers are aware of the potential impact of this discrepancy on the comprehensiveness of our research findings. Furthermore, to include a broader range of cases, we categorized staging into localized, regional, and distant stages. The regional category encompasses both local advancement and pelvic lymph node metastases. However, the data we extracted does not sufficiently identify which patients within this group have lymph node metastases. By the way, despite this limitation, it’s important to note that the current treatment approach for PCa patients classified as regional stage—whether it involves local progression or lymph node metastasis—tends to be similar, involving either adjuvant hormone therapy or radiation therapy. Moreover, the absence of data on urinary incontinence represented a significant shortcoming in our research. We were aware that both RP and TURP carry the risk of urinary incontinence. How the rate of urinary incontinence after TURP followed by RP affects patients’ quality of life is also of great importance. In the future, we hope to use our own data to fill this gap and address this shortcoming. In addition, our cases differed from the iPCa patients diagnosed by TURP; these iPCa patients were generally in the early stage, while a considerable number of our patients had advanced PC. In addition, we included a few patients who offered RT, and the benefit of RT was not assessed and compared with the long-term survival benefit of patients with RP. Furthermore, the SEER database does not record functional data such as urinary incontinence, sexual function, and quality of life. Thus, we could not compare these items in this study’s two groups.

## Conclusions

We presented this retrospective population-based, matched study and found that RP conferred survival benefits for PCa patients diagnosed with TURP, especially because it reduced the risk of CSM. This kind of benefit was seen even in old aged (> 75ys.) patients with advanced PCa. However, it may be needed to offer systemic therapy for advanced PCa patients.

### Electronic supplementary material

Below is the link to the electronic supplementary material.

Supplementary Material 1

## Data Availability

Data is provided within the supplementary information files.
